# Dietary Supplementation With Leucine or in Combination With Arginine Decreases Body Fat Weight and Alters Gut Microbiota Composition in Finishing Pigs

**DOI:** 10.3389/fmicb.2019.01767

**Published:** 2019-08-13

**Authors:** Chengjun Hu, Fengna Li, Yehui Duan, Yulong Yin, Xiangfeng Kong

**Affiliations:** ^1^Hunan Provincial Key Laboratory of Animal Nutritional Physiology and Metabolic Process, Key Laboratory of Agro-Ecological Processes in Subtropical Region, National Engineering Laboratory for Pollution Control and Waste Utilization in Livestock and Poultry Production, Institute of Subtropical Agriculture, Chinese Academy of Sciences, Changsha, China; ^2^Guangdong Provincial Key Laboratory of Animal Nutrition Control, Institute of Subtropical Animal Nutrition and Feed, College of Animal Science, South China Agricultural University, Guangzhou, China

**Keywords:** arginine, colon, dietary supplementation, glutamic acid, leucine, microbiota, short-chain fatty acid

## Abstract

Obesity was associated with change in gut microbiota composition and their metabolites. We investigated the effects of dietary supplementation with leucine (Leu) in combination with arginine (Arg) or glutamic acid (Glu) on body fat weight, composition of gut microbiota, and short-chain fatty acids (SCFAs) concentration in the colon. Forty-eight Duroc × Large White × Landrace pigs with an initial body weight of 77.08 ± 1.29 kg were randomly assigned to one of the four groups (12 pigs per group). The pigs in the control group were fed a basal diet supplemented with 2.05% alanine (isonitrogenous control, BD group), and those in the three experimental groups were fed a basal diet supplemented with 1.00% Leu + 1.37% alanine (Leu group), 1.00% Leu + 1.00% Arg (Leu_Arg group), or 1.00% Leu + 1.00% Glu (Leu_Glu group). We found that dietary supplementation with Leu alone or in combination with Arg decreased (*p* < 0.05) body fat weight, and increased (*p* < 0.05) colonic propionate and butyrate concentrations compared to the BD group. The mRNA expression levels of genes related to lipolysis increased (*p* < 0.05) in the Leu or Leu_Arg group compared to the BD group. Negative relationships (*p* < 0.05) were observed between body fat weight, colonic propionate, and butyrate concentrations. Compared to the BD group, the abundance of *Actinobacteria* was higher (*p* < 0.05) in the Leu group, and that of *Clostridium_sensu_stricto*_1, *Terrisporobacter*, and *Escherichia-Shigella* were higher in the Leu_Arg group. The abundance of *Deinococcus-Thermus* was negatively correlated (*p* < 0.05) with body fat weight, and was positively correlated (*p* < 0.05) with butyrate, isovalerate, propionate, and isobutyrate concentrations, and that of *Cyanobacteria* was positively correlated (*p* < 0.05) with butyrate, propionate, and isobutyrate concentrations. In conclusion, these findings suggest that decreased body fat weight in pigs can be induced by Leu supplementation alone or in combination with Arg and is associated with increased colonic butyrate and propionate concentrations. This provides new insights for potential therapy for obesity.

## Introduction

Over the past few decades, obesity has increased from 16.8% in 2007–2008 to 18.5% in 2015–2016 among youth, and from 33.7% in 2007–2008 to 39.6% among adults (Hales et al., 2018). Obesity exerts a negative impact on human health, including causing insulin resistance, diabetes mellitus, cancer, inflammation, sleep apnea, and other chronic diseases (Zhang et al., 2018). It causes more than 3.4 million deaths worldwide (Lim et al., 2013). Although obesity is the one of most important public health challenge (Simmonds et al., 2016), there are few types of medication available for preventing and treating this disease.

The amino acid leucine (Leu) is a substrate for protein synthesis and is involved in the regulation of fat metabolism (Yao et al., 2016). Studies have confirmed that Leu has the potential to prevent and treat obesity. For instance, Leu supplementation with 50% food restriction results in lower body fat in rats than those subjected to the same 50% food restriction (Donato et al., 2006). Increased dietary Leu intake reduces diet-induced obesity and improves glucose metabolism (Zhang et al., 2007). In addition, Leu treatment improves mitochondrial biogenesis, fatty acid oxidation, insulin sensitivity, and glucose metabolism in diet-induced obesity in mice (Guo et al., 2010; Li et al., 2012). Arginine (Arg) and glutamic acid (Glu) also play important roles in fat metabolism. Dietary supplementation with 1% Arg reduces body fat accumulation in pigs (Tan et al., 2009), and supplementation with 0.24% L-Arg-HCl in drinking water reduces fat accretion in adult ZDF rats (Wu et al., 2007). Our previous study showed that dietary supplementation with 1.00% Glu decreased back fat thickness in finishing pigs (Hu et al., 2017a), indicating that body fat accumulation declined with Glu treatment. Although studies have demonstrated that Arg and Glu play vital roles in preventing obesity, the effects of dietary supplementation with Leu in combination with Arg or Glu on fat accumulation are still unknown.

Changes in the gut-microbiota community have been proposed as possible causes of obesity. There are distinct differences at the phylum level in the microbiota community between obese and lean subjects; obese subjects have lower bacterial diversity, and different metabolic pathways (Turnbaugh et al., 2009). In addition, inoculation of the microbiota in adult obese mice into germ free mice increases the total body fat in germ free mice (Turnbaugh et al., 2006), suggesting gut microbiota as a contributing factor to obesity. Short-chain fatty acids (SCFAs) are produced by the microbiota in the large bowel *via* fermentation of carbohydrates and amino acids (Rios-Covian et al., 2016) and have key roles in anti-obesity. For instance, butyrate protects against diet-induced obesity by reducing food intake and increasing energy expenditure (Gao et al., 2009; Lin et al., 2012), and dietary SCFA supplementation prevented and reversed high-fat diet-induced obesity in mice by decreasing peroxisome proliferator activated receptor-γ expression and activity (den Besten et al., 2015). Amino acids in the large intestine are the substrate for SCFAs. These findings motivated us to investigate whether Leu in combination with Arg or Glu alters SCFAs concentrations in the colon. The pig is one of the most commonly used model animals in biomedical studies on human obesity (Houpt et al., 1979). Therefore, our objective was to investigate the effects of dietary supplementation with Leu in combination with Arg or Glu on body fat weight, composition of the gut microbiota, and SCFA concentration in the colons in pigs.

## Materials and Methods

### Ethics Statement

The protocol for this study was approved by the Committee on the Ethics of Animal Experiments of the Institute of Subtropical Agriculture, Chinese Academy of Sciences under ethic approval number ISA-2017-023, and was conducted in accordance with the recommendations of the Guide for the Care and Use of Laboratory Animals of the Institute of Subtropical Agriculture, Chinese Academy of Sciences.

### Animals and Experimental Treatments

Forty-eight Duroc × Large White × Landrace pigs were selected and randomly assigned to one of four groups (12 pigs per group). The average body weight of the pigs used for this study was 77.09 kg. The pigs in the control group were fed a basal diet supplemented with 2.05% alanine (isonitrogenous control, BD group), and those in the three experimental groups were fed a basal diet supplemented with 1.00% Leu + 1.37% alanine (Leu group), 1.00% Leu + 1.00% Arg (Leu_Arg group), or 1.00% Leu + 1.00% Glu (Leu_Glu group). The basal diet was formulated on the basis of nutrient requirements established by the National Research Council (2012) (Supplementary Table S1). The amino acids Ala, Leu, Arg, and Glu were obtained from Wuxi Jinghai Amino Acid Co., Ltd. (Wuxi, China). The pigs were housed in cages (3.5 m × 5.0 m) and fed for 60 days. The pigs had 24 h access to feed and water. The final body weight of pigs was not affected by diet treatment.

### Sample Collection and Body Fat Weight Determination

At the end of the trial, blood samples were obtained from the jugular vein of fasted pigs using 10 ml centrifuge tubes containing sodium heparin; samples were centrifuged at 3,000 ×*g* for 15 min to recover the plasma. After that, the pigs were slaughtered by electric shock (120 V, 200 Hz) and exsanguination. After removing the head, feet, tail, and internal organs, the carcass was cut into right and left parts longitudinally. The body fat was removed from the right side of the carcass and then weighed. The fat between the sixth and seventh ribs were immediately collected from the right side of the carcass, and snap-frozen in liquid nitrogen (approximately, 10 g per sample) and then stored at −80°C for mRNA analysis. The colon was quickly separated and the luminal contents were collected in sterile tubes and stored at −80°C for laboratory analysis.

### RNA Extraction and Complementary DNA Synthesis

Total RNA was extracted from the fat tissue (approximately 50 mg per sample) using TRIzol reagent (Life Technologies, Carlsbad, CA, USA). The RNA concentration and 260:280 nm ratio of each sample was measured using the NanoDrop^®^ ND-1000 instrument (Thermo Fisher, Wilmington, DE, USA). RNA integrity was determined using 1% agarose gel electrophoresis. All RNA samples examined in this study showed the 5S, 18S, and 28S rRNA bands. Complementary DNA (cDNA) was synthesized from 1,000 ng RNA in a 20 μl reaction volume using a PrimeScript^®^ first strand cDNA synthesis kit (Takara, Osaka, Japan), and stored at −80°C until further analysis.

### Real-Time Polymerase Chain Reaction Analysis

Primers selected for polymerase chain reaction (PCR) analyses were designed using Primer 3^1^ and are listed in Supplementary Table S2. Total reaction volumes (10 μl) comprised of 2 μl cDNA template solution, 5 μl SYBR Green PCR master mix (Thermo Fisher Scientific, Inc., Waltham, MA, USA), 2.2 μl water, and 0.4 μl of each primer. The relative expression levels of genes were determined using the ABI 7900HT system (Applied Biosystems, Carlsbad, CA, USA) and three replicates per biological sample. The RT-PCR program included a 10-min incubation at 95°C, followed by 40 cycles of denaturation for 15 s at 95°C and annealing and extension for 20 s at 60°C. A melting curve program (60–99°C with a heating rate of 0.1°C/s) and fluorescence measurement was performed to generate melting curves for each sample, check primer specificity, and ensure the purity of PCR products. The gene glyceraldehyde-3-phosphate dehydrogenase (*GAPDH*) was used to normalize the mRNA levels of the selected genes. The relative expression level of mRNA was calculated according to the following formula (Hu et al., 2017b): R = 2^−∆∆Ct(sample-control)^, where ∆∆Ct(sample-control) = (Ct_target gene_ – Ct*_GAPDH_*) treated – (Ct_target gene_ – Ct*_GAPDH_*) control.

### Plasma Biochemical Parameters

Plasma total cholesterol (TC), triglycerides (TG), low density lipoprotein-cholesterol (LDL-C), high density lipoprotein-cholesterol (HDL-C), and lipase were measured using a biochemical analytical instrument TBA-120FR (Toshiba, Otawara-shi, Japan) and respective commercial assay kits (Yonghe-Yangguang Science and Technology Co., Ltd., Changsha, China) according to the manufacturers’ instructions.

### Metabolite Concentrations in Colonic Contents

The SCFAs, including acetate, propionate, butyrate, isobutyrate, pentanoate, and isopentanoate were analyzed by gas chromatography as described previously (Ji et al., 2018). Bioamines, including putrescine, cadaverine, spermidine, spermine, and tyramine were measured by high-performance liquid chromatography as described previously (Ji et al., 2018).

### DNA Extraction and 16S rRNA Gene Sequencing

Total microbial DNA was extracted from colonic content samples (*n* = 6 per group) using the HiPure Stool DNA kit B (Magen, Shanghai, China). The DNA concentration was measured using the NanoDrop^®^ ND-1000 instrument (NanoDrop Technologies Inc., USA). The V3-V4 region of the 16S rRNA gene was amplified using the universal primers 515F (5′-GTGCCAGCMGCCGCGGTAA-3′) and 806R (5′-GGACTACHVGGGTWTCTAAT-3′) (Zeng et al., 2017). The PCR was performed in a total volume of 20 μl comprising 1 μl of DNA, 2 μl of deoxyribonucleotide triphosphate, 4 μl of 5-fold FastPfu buffer (TransGen Biotech, China), 0.4 μl of FastPfu polymerase (TransGen Biotech, China), 0.8 μl (5 μM) of each primer, and 11 μl ddH_2_O. The PCR program included a 3-min incubation at 95°C, followed by 27 cycles of denaturation at 95°C for 30 s, and annealing and extension at 55°C for 30 s and at 72°C for 45 s. The amplified PCR product was clearly identified using 1.2% agarose gels. Amplicons were then extracted from the agarose gels and purified using the SanPrep DNA Gel Extraction kit (Sangon Biotech, Shanghai, China). Purified amplicons were then subject to paired-end sequencing on the Illumina MiSeq platform (Illumina, Sand Diego, CA, USA) according to the manufacturer’s instructions; this was performed by a commercial service provider (Shanghai Majorbio Bio-pharm Technology Co., Ltd., Shanghai, China).

### Bioinformatics Analysis

Raw Illumina fastq files were quality-filtered, de-multiplexed, and analyzed using Trimmomatic (v.0.30) (Bolger et al., 2014) and FLASH (v.1.2.11) (Magoc and Salzberg, 2011) software packages; they were filtered to eliminate adapters and low-quality reads to obtain clean reads; overlapped paired-end reads were then merged to create tags. The main steps were as follows: (1) the bases with a trailing quality score < 20 were removed; (2) 300 bp reads at any site receiving an average quality score < 20 over a 50-bp sliding window were truncated, and truncated reads of <50 bp were removed; (3) merged reads with a mismatch ratio in the overlapping regions of <0.2 were removed, whereas the sequences that overlapped by >10 bp were assembled according to their overlap sequence; and (4) reads with primer mismatches >2 and with barcode mismatches >0 were removed. Tags were clustered into operational taxonomic units (OTUs) with sequence similarity of 97% using USEARCH (v7.0.1090) (Edgar, 2013). Representative OTU sequences were taxonomically classified using the Ribosomal Database Project (RDP) Classifier based on the Greengene (V201305) reference database. Alpha diversity values for colonic bacterial communities were estimated using the ACE, the bias-corrected Chao richness estimator, Shannon, and Simpson indices. Partial least squares discriminant analysis (PLS-DA) was used to analyze the unadjusted means of OTU-level microbial abundances. The 16S rRNA gene sequence was submitted to the NCBI Sequence Read Archive database under accession numbers SRR9672929–SRR9672950.

### Statistical Analyses

Total fat weight, colonic metabolite, and alpha diversity indices were analyzed using one-way analysis of variance (ANOVA) and Duncan’s multiple-range *post hoc* test in SPSS software (v20.0; SPPS Inc., Chicago, IL, USA). The relative species abundances and overall composition (at phyla and genera level) of gut microbial communities were analyzed using the Kruskal-Wallis test. Pearson’s correlation coefficient was used to assess the relationships between body fat weight, SCFA levels, and the relative abundances of phyla. LEfSe was used to identify different taxa microbes among lines using default parameters. Results were expressed as means ± SEM. Differences were considered statistically significant at *p* less than 0.05.

## Results

### Body Fat Weight and Plasma Analysis

**Figure 1:**
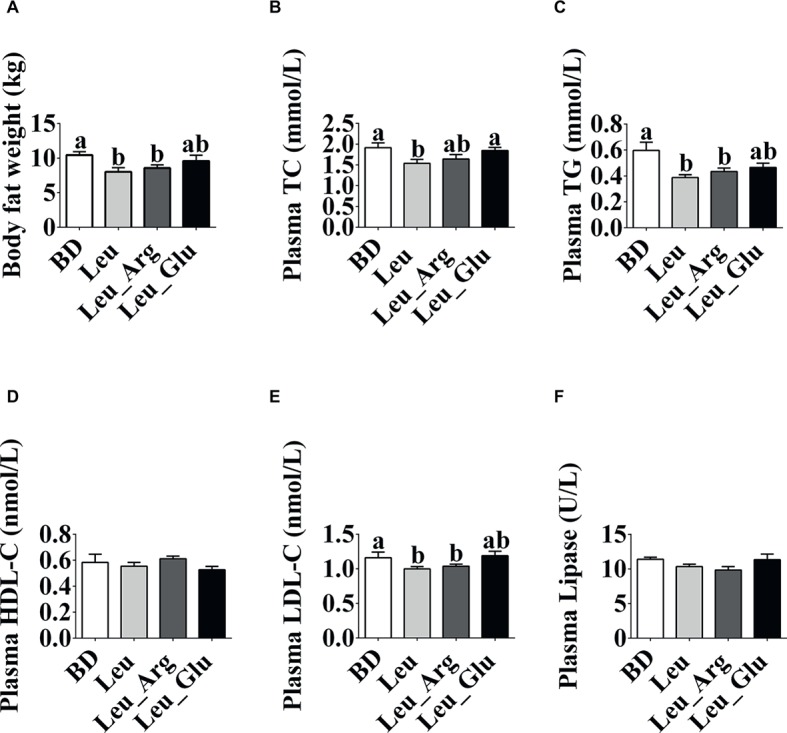
Phenotype changes under dietary Leu, Arg, and Glu treatment. **(A)** Body fat weight. **(B-F)** Plasma TC, TG, HDL-C, LDL-C, and lipase concentrations. Data represent the means ± SEM. ^a,b^indicate statistically significant differences (*p* < 0.05).

As shown in Figure 1, dietary supplementation with Leu or Leu_Arg reduced (*p* < 0.05) body fat weight in finishing pigs, whereas supplementation with Leu_Glu did not (*p <* 0.05) affect body fat weight, relative to the BD group.

The plasma TG and LDL-C concentrations were lower (*p* < 0.05) in the Leu and Leu_Arg groups than in the BD group. The plasma TC concentration was lower (*p* < 0.05) in the Leu group than in the BD or Leu_Glu group.

### Expression of Fat Metabolism-Related Genes

**Figure 2:**
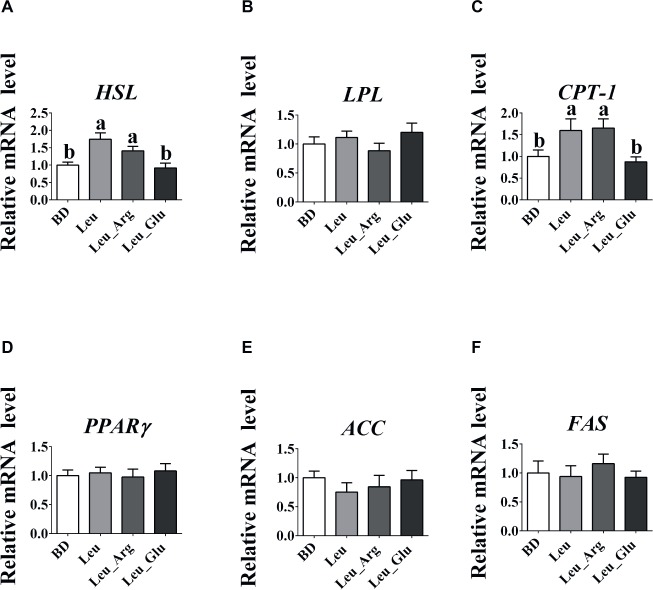
The mRNA expression levels of fat-metabolism genes in the subcutaneous adipose tissues. The relative mRNA expression levels of HSL **(A)**, LPL **(B)**, CPT-1 **(C)**, PPARγ **(D)**, ACC **(E)**, and FAS **(F)**. Data represent the means ± SEM. ^a–c^indicate statistically significant differences (*p* < 0.05).

As shown in Figure 2, no significant differences (*p* > 0.05) were observed in the mRNA expression levels of lipoprotein lipase (*LPL*), peroxisome proliferator-activated receptor γ (*PPARγ*), acetyl-coA carboxylase (*ACC*), and fatty acid synthase (*FAS*) among the four dietary groups. The mRNA expression level of hormone-sensitive lipase (*HSL*) was higher (*p* < 0.05) in the Leu and Leu_Arg groups than in the BD group, and that of carnitine palmitoyl transferase-I (*CPT*-1) was higher (*p* < 0.05) in the Leu_Arg group than in the BD or Leu_Glu group.

### Concentrations of Short-Chain Fatty Acids and Bioamines in Colonic Contents

**Figure 3:**
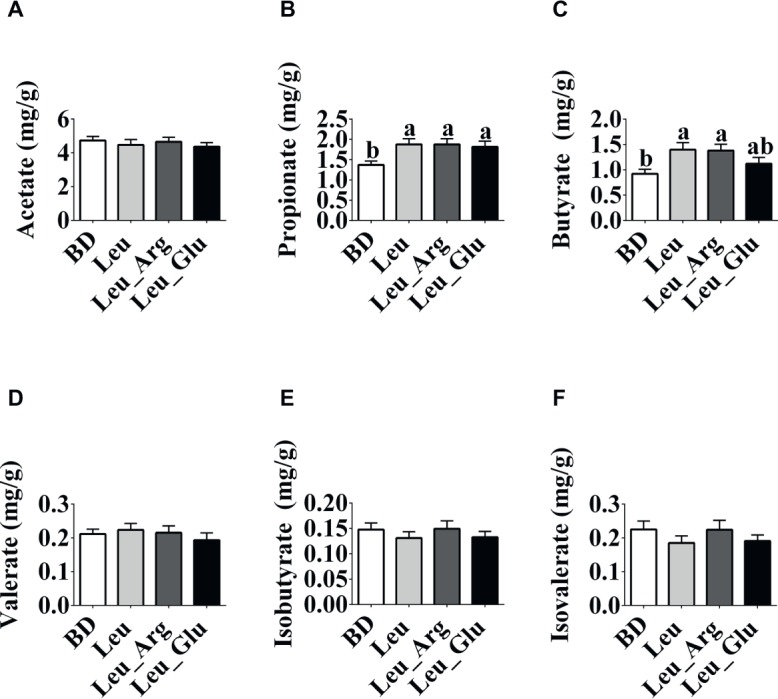
The SCFAs concentrations in the colonic contents in finishing pigs. **(A-F)** Colonic acetate, propionate, butyrate, valerate, isobutyrate, and isobutyrate concentrations, respectively. Data represent the means ± SEM. ^a,b^indicate statistically significant differences (*p* < 0.05).

**Figure 4:**
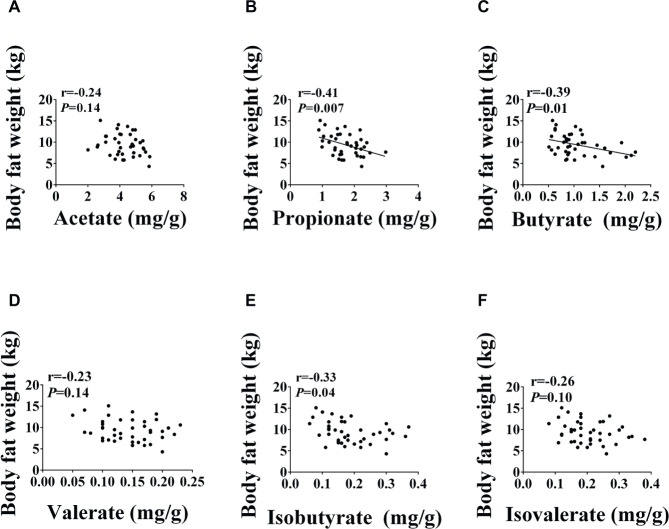
Relationship between SCFAs and body fat weight. Pearson correlations were used to determine the association between body fat weight and colonic acetate **(A)**, propionate **(B)**, butyrate **(C)**, valerate **(D)**, isobutyrate **(E)**, and isobutyrate **(F)** concentrations.

As shown in Figure 3, the concentrations of acetate, valerate, isobutyrate, and isovalerate were not affected (*p* < 0.05) by diet. The concentrations of propionate and butyrate were significantly elevated (*p* < 0.05) in the Leu and Leu Arg groups relative to the BD group. Moreover, the concentration of propionate was higher (*p* < 0.05) in the Leu_Glu group than in the BD group (*p* < 0.05). Correlations between SCFAs concentrations and body fat weight are presented in Figure 4. Significant negative correlations (*p* < 0.05) were observed between body fat weight and the colonic concentrations of propionate, butyrate, and isobutyrate.

**Figure 5:**
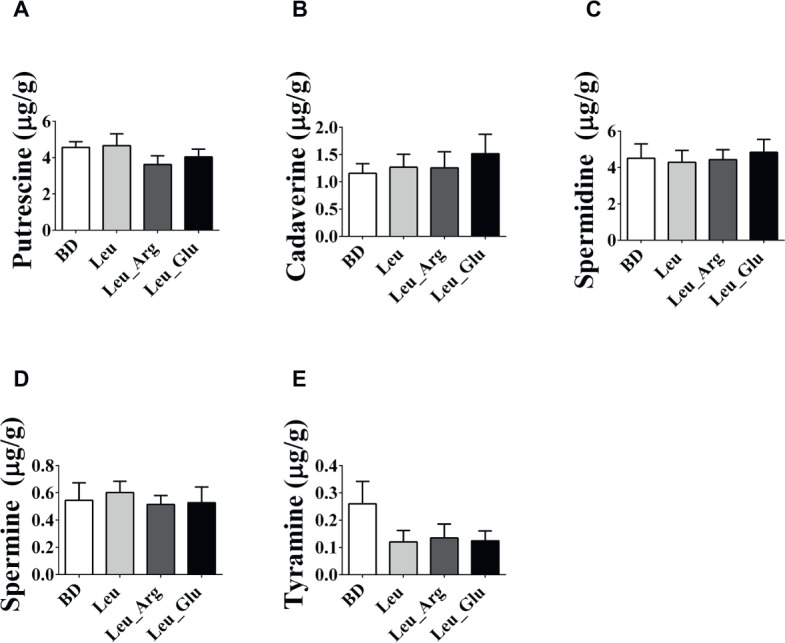
The bioamines concentrations in the colonic contents in finishing pigs. **(A-E)** Colonic putrescine, cadaverine, spermidine, spermine, and tyramine concentrations, respectively. Data represent the means ± SEM.

As shown in Figure 5, no differences (*p* < 0.05) were observed in the concentrations of putrescine, cadaverine, spermidine, spermine, and tyramine among the dietary treatment groups.

### Diversity of Colonic Bacterial Communities

**Figure 6:**
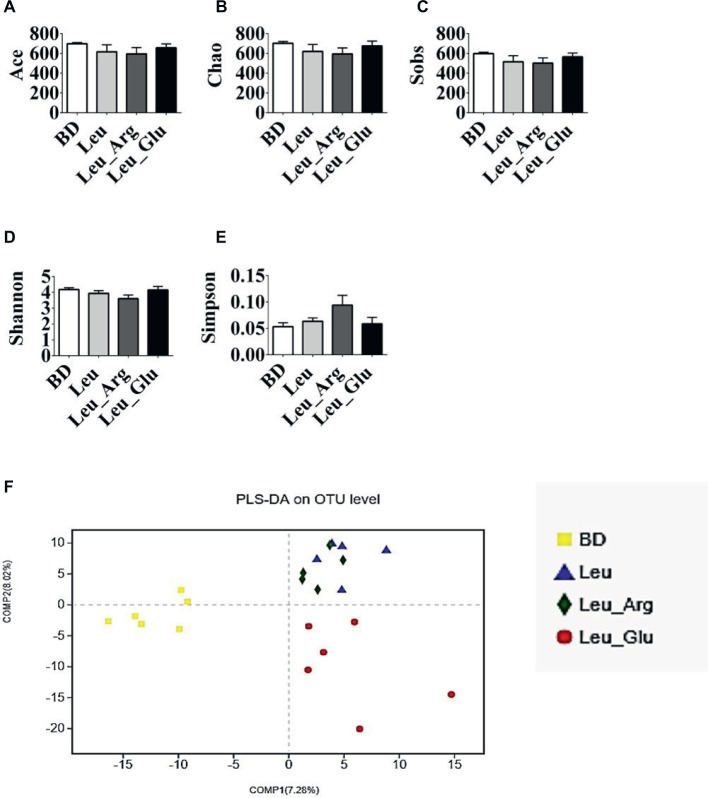
Alpha diversity of colonic bacterial community of finishing pigs with different dietary treatment. **(A–E)** The bacterial diversity estimated by Ace, Chao, Sob, Shannon, and Simpson indexes. **(F)** Partial least squares discrimination analysis (PLS-DA) of colonic bacterial community.

After size filtering, quality control, and chimera removal, 874,597 valid sequences were obtained, with an average of 39,754 sequences per colonic sample (Supplementary Table S3). These sequences were assigned to 1,098 OTUs. Overall, 971, 918, 919, and 969 OTUs were obtained from pigs in the BD, Leu, Leu_Arg, and Leu_Glu dietary treatments, respectively (Supplementary Table S3). Dietary supplementation with Leu, Leu_Arg, or Leu_Glu did not affect (*p* < 0.05) the ACE, Chao, Sobs, Shannon, and Simpson indices of the sampled bacterial communities (Figures 6A–E). According to PLS-DA, samples from the Leu and Leu_Arg groups were clustered together (Figure 6F).

### Colonic Bacterial Community Structure

**Figure 7:**
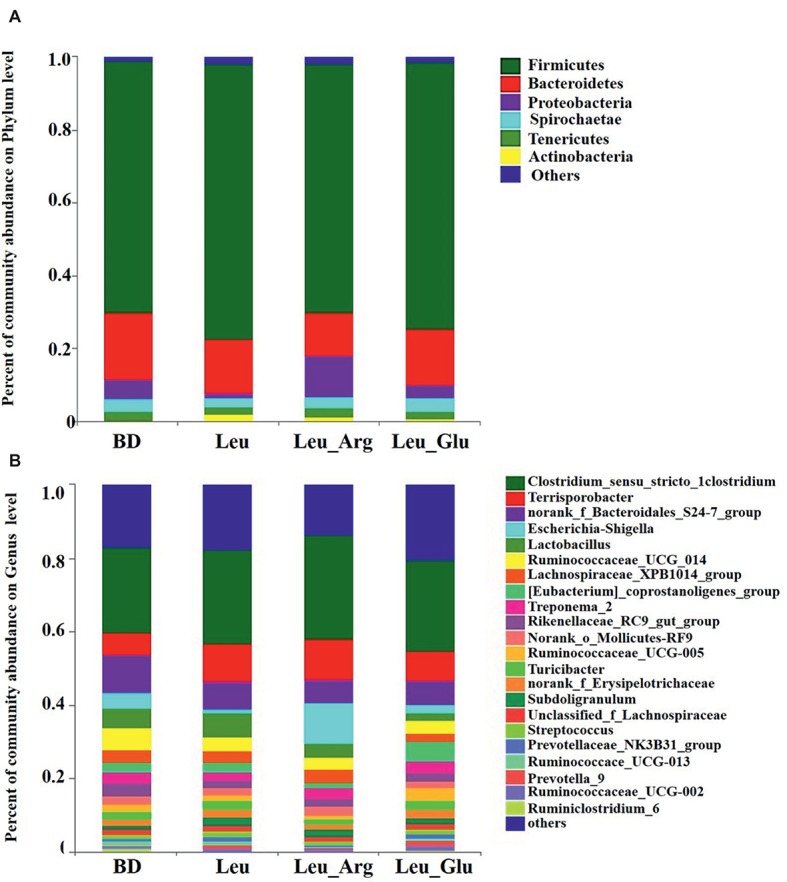
Colonic bacterial community structure of finishing pigs with different dietary treatment. Distribution of colonic bacteria at phylum **(A)** and genera **(B)** levels in finishing pigs. Phylum and genera with proportion less than 0.01 are not listed.

**Figure 8:**
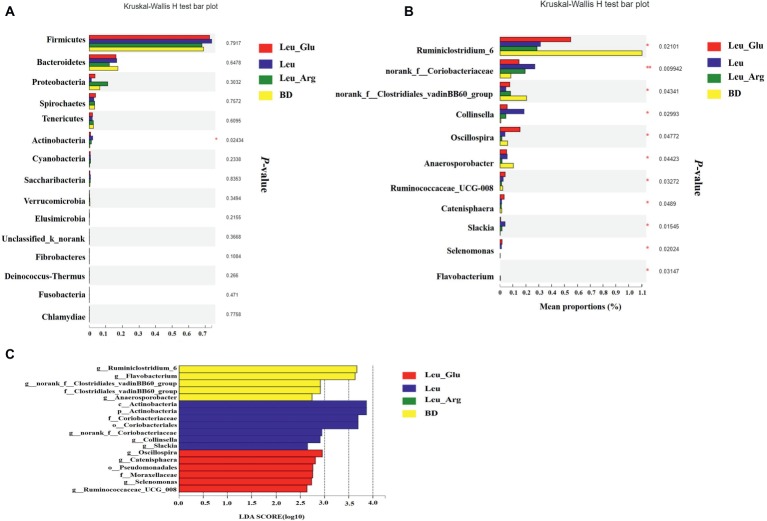
Taxonomic differences of colonic microbiota among the four groups. Comparison of relative abundance at the phylum **(A)** and genus **(B)** levels between the four groups. In genes levels, only data of which differences with *p* less than 0.05 are shown. All the differences were analyzed using Mann-Whitney *U* test; ^∗^*p <* 0.05; ^∗∗^*p <* 0.01. **(C)** Linear discriminant analysis (LDA) scores for the enriched microbiota.

The most dominant phyla in the bacterial communities (comprising >1% of the community) were *Firmicutes*, *Bacteroidetes*, *Proteobacteria*, *Spirochaetes*, *Tenericutes*, and *Actinobacteria* (Figure 7A), comprising >97% of the total colonic bacteria found in gut samples. *Firmicutes* was observed at highest abundance in the Leu group (73.85%). The abundance of *Bacteroidetes* was lower, while that of *Proteobacteria* was higher, in the Leu_Arg group than in the BD group. The abundance of *Actinobacteria* was higher (*p* < 0.05) in the Leu group than in the BD group (Figure 8A).

Figure 7B shows the distribution of the abundances of the bacterial genera (>1%) among the four treatment groups. The abundances of *Clostridium_sensu_stricto_*1, *Terrisporobacter*, and *Escherichia-Shigella* were highest in the Leu_Arg group, and that of *Lactobacillus* was highest in the Leu group. Dietary supplementation with Leu or Leu_Arg reduced (*p* < 0.05) the abundances of *Ruminiclostridium_6* and *norank_f_Clostridiales_vadinBB60_group* (Figure 8B). The abundances of *norank_f_Coriobacteriaceae* and *Collinsella* were higher (*p* < 0.05) in the Leu group than in the BD group.

To further compare the taxonomic difference among the four groups, LEfSe analysis was used to assess the differential abundance of bacterial taxa (Figure 8C): six bacterial biomarkers were differentially abundant among the four groups. *Actinobacteria* and *Coriobacteriales* were the dominant microbes in the Leu group.

### Relationships Between Bacterial Community Composition, Body Fat Weight, and Metabolite Concentrations

**Figure 9:**
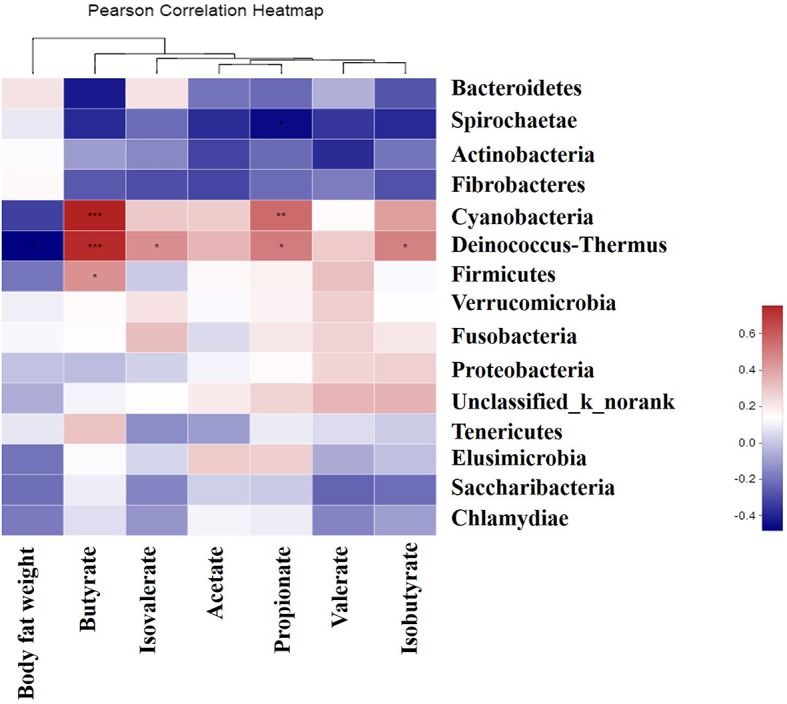
Correlations between the genera, body fat weight, and the colonic metabolite concentrations. Asterisk in blue grid indicates a negative correlation between the abundance of the genera, body fat weight, and metabolites concentrations, whereas asterisk in the crimson grid represents a positive correlation. *0.01 < *p* ≤ 0.05, **0.001 < *p* ≤ 0.01,***p ≤ 0.001.

As shown in Figure 9, the abundance of *Deinococcus-Thermus* was negatively correlated (*p* < 0.05) with body fat weight, and was positively correlated (*p* < 0.05) with butyrate, isovalerate, propionate, and isobutyrate concentrations. The abundance of *Cyanobacteria* was positively correlated (*p* < 0.05) with butyrate, propionate, and isobutyrate concentrations, and that of *Firmicutes* was positively correlated (*p* < 0.05) with butyrate concentration. In addition, the abundance of *Spirochaetae* was negatively correlated (*p* < 0.05) with propionate concentration.

## Discussion

To the best of our knowledge, the effects of supplementation with Leu in combination with Arg or Glu on fat accumulation have not been previously reported. Therefore, we investigated the effects of Leu in combination with Arg or Glu on body fat weight, and determined whether body fat weight was associated with colonic microbial composition and SCFAs. We found that dietary supplementation with Leu decreased body fat weight, consistent with Vianna et al. (2012), who reported that long-term Leu supplementation reduces fat mass gain in rats (Vianna et al., 2012). Further, Leu in combination with Arg, but not with Glu, reduced body fat weight, revealing synergistic effects between Leu and Arg. Arginine can reduce fat accumulation in animals (Tan et al., 2009). Therefore, it is not surprising that body fat weight decreased following dietary supplementation with both Leu and Arg. We detected a possible antagonism between Leu and Glu. Our previous study showed that dietary supplementation with 1% Glu reduced back fat thickness in finishing pigs (Hu et al., 2017a,b), indicating that fat accumulation was attenuated with Glu treatment. Here, in contrast, we found that Glu supplementation reversed the effects of Leu on fat accumulation, as well as plasma TG and TC concentrations. Fat accumulation is related to the process of lipogenesis and lipolysis. Therefore, we analyzed genes related to lipogenesis and lipolysis. Our results suggest that the reduced body fat weight in the Leu and Leu_Arg groups might arise from the upregulated expression of *HSL* and *CPT1* in the adipose tissue. We found that genes involved in lipogenesis (*PPAR*γ, *ACC*, and *FAS*) were not affected by diet supplementation, whereas genes associated with lipolysis, including *HSL* and *CPT-1*, were elevated in the Leu and Leu_Arg groups. Hormone-sensitive lipase is responsible for catalyzing the hydrolysis of triacylglycerols in adipose tissue (Enevoldsen et al., 2001), and CPT-1 is mainly responsible for transferring cytosolic long-chain fatty acyl CoA into the mitochondria for oxidation. Dietary supplementation with Leu or Arg has been shown to increase fatty acid oxidation in fat (Jobgen et al., 2006; Chen et al., 2012; Zemel and Bruckbauer, 2012), suggesting that the reduced body fat weight in the Leu and Leu_Arg groups was associated with fatty oxidation.

Obesity is associated with changes in composition, diversity and function of the gut microbiota. Therefore, gut microbiota composition in colon were determined. Our finding found that *Firmicutes* were most abundant in the Leu group, which is consistent with prior findings of elevated *Firmicutes* abundance and reduced *Bacteroidetes* abundance in obese mice (Ley et al., 2005) and in obese humans (Chakraborti, 2015). In contrast, Duncan et al. (2008) did not observe any difference in the abundance of *Bacteroidetes* and *Firmicutes* in the feces of lean and obese humans. It is clear that further research is needed to clarify the relationships between *Firmicutes*, *Bacteroidetes,* and obesity. Arg supplementation in mice is known to increase the abundance of *Bacteroidetes* and reduce that of *Firmicutes* in the jejunum and ileum (Ren et al., 2014), and Glu supplementation in pigs increases *Bacteroidetes* and *Peptostreptococcus* abundance in ileum (Feng et al., 2015). The abundance of *Actinobacteria* was highest in Leu group, in line with Pedersen et al. (2013) who reported that a higher abundance of *Actinobacteria* was observed in the cecal microbiota of lean Göttingen minipigs. However, elevated abundance of *Actinobacteria* has been demonstrated in obese humans (Turnbaugh et al., 2009). This discrepancy in the results of the present study and previous studies might be explained by differences between the species and diets used. We do not know of any possible reason to explain our finding that the abundance of *Deinococcus-Thermus* was negatively correlated with body fat weight, and was positively correlated with butyrate, isovalerate, propionate, and isobutyrate concentrations. However, the role of *Deinococcus-Thermus* in the alterations of these phenotypes remains unknown.

The SCFAs produced by the colonic microbiota provide 60–70% of the energy needs of colonic cells (Topping and Clifton, 2011). Of the SCFAs, butyrate is the major source of energy for the colonic epithelium. Colonic concentrations of SCFAs are associated with obesity: total SCFA concentration was significantly higher in obese humans than lean humans (Rahat-Rozenbloom et al., 2014), and elevated colonic propionate prevents weight gain in overweight adult humans (Chambers et al., 2015). We observed that dietary supplementation with Leu alone or in combination with Arg increased colonic propionate and butyrate concentrations, and negative correlations were observed between body fat weight and the concentrations of both propionate and butyrate. Consistent with these findings, studies in rodents have found that butyrate and propionate prevent diet-induced obesity and insulin resistance (Lin et al., 2012; Hong et al., 2016; Weitkunat et al., 2016; Wang et al., 2018). Further, elevated butyrate and propionate levels are known to reduce body fat mass mainly causing reduced intake of food or energy (Lin et al., 2012; Chambers et al., 2015). It has also been suggested that inhibition of adipose tissue accumulation is associated with elevated propionate produced by the gut microbiota; this may be because propionate increases energy expenditure (Kimura et al., 2011). In contrast, reduced body fat mass has been shown to be associated with increased cecal propionate, although no difference was observed in energy intake (Liou et al., 2013). We found that dietary supplementation with Leu alone or in combination with Arg reduced body fat weight and increased butyrate and propionate concentrations in the colon; however, in our previous study, feed intake was not affected by these amino acids (Hu et al., 2019). The discrepancy between the present study and our previous study might be explained by the fact that pigs consume more feed than mice.

## Conclusions

We found that dietary supplementation with Leu alone or in combination with Arg reduced body fat weight and increased the expression of genes involved in lipolysis in adipose tissue; it raised colonic butyrate and propionate concentrations, which were associated with reduced body fat weight. These findings provide new insight into the role of Leu in combination with Arg in preventing obesity. However, the mechanisms whereby Leu and Arg contribute to butyrate and propionate formation in the colon remain unclear. It is also unknown whether decreased body fat weight attributes to elevated colonic butyrate and propionate concentrations.

## Author Contributions

CH and XK contributed to the study design, conducted the animal experiments, and wrote the manuscript. CH executed the lab analysis. YD and FL performed the statistical analysis. XK and YL revised the paper. All authors carefully read and approved the final revision of the manuscript.

### Conflict of Interest Statement

The authors declare that the research was conducted in the absence of any commercial or financial relationships that could be construed as a potential conflict of interest.

## Supplementary Material

The Supplementary Material for this article can be found online at: https://www.frontiersin.org/articles/10.3389/fmicb.2019.01767/full#supplementary-material

## Footnotes

^1^http://bioinfo.ut.ee/primer3-0.4.0/
