# Supplementary material for: Dietary Supplementation With Leucine or in Combination With Arginine Decreases Body Fat Weight and Alters Gut Microbiota Composition in Finishing Pigs
Source: Front Microbiol. 2019 Aug 13;10:1767. doi: 10.3389/fmicb.2019.01767 (PMC6700229; doi:10.3389/fmicb.2019.01767)
Supplement: Supplementary file 1 [file Table_1.docx]

Supplementary Material

**Table S1.** Composition and nutrient levels of the basal diets (as-fed basis)

| Ingredient | % | Chemical composition^2^ | % |
| --- | --- | --- | --- |
| Corn | 76.3 | DE, MJ/kg | 14.26 |
| Soybean meal | 16.5 | CP, % | 13.04 |
| Soybean oil | 0.56 | CF, % | 2.42 |
| Corn starch | 2.44 | EE, % | 5.94 |
| CaHPO_4_ | 0.65 | Ca, % | 0.62 |
| Calcium carbonate | 1.08 | Total P, % | 0.42 |
| Salt | 0.43 | AP, % | 0.20 |
| Lys | 0.16 | Asp+Asn | 1.73 |
| Thr | 0.08 | Thr | 0.79 |
| Try | 0.05 | Ser | 0.80 |
| Premix^1^ | 1.75 | Glu+Gln | 3.06 |
| Total | 100 | Gly | 0.95 |
|  |  | Ala | 1.09 |
|  |  | Val | 0.83 |
|  |  | Met | 0.24 |
|  |  | Ile | 0.79 |
|  |  | Leu | 1.50 |
|  |  | Tyr | 0.48 |
|  |  | Phe | 0.77 |
|  |  | His | 0.62 |
|  |  | Lys | 0.85 |
|  |  | Arg | 1.01 |
|  |  | Pro | 1.56 |
|  |  | Cys | 0.28 |

^1^Premix provided for 1 kg of complete diet: Cu as copper sulfate,10 mg; Fe as iron sulfate, 100 mg; Se as sodium selenite, 0.30 mg; Zn as zinc oxide, 100 mg; Mn as manganese oxide, 10 mg; vitamin D_3_, 386 IU; vitamin A as retinyl acetate, 3 086 IU; vitamin E as D-atocopherol, 15.4 IU; vitamin K as menadione sodium bisulfate, 2.3 mg; vitamin B_2_, 3.9 mg; calcium pantothenate, 15.4 mg; niacin, 23 mg; and vitamin B_12_, 15.4 mg.

^2^Analyzed values.

**Table S2.** Primers used for real-time PCR

| Genes | Primers | Sequences (5^，^to 3^，^) | Accession Number | Size (bp) |
| --- | --- | --- | --- | --- |
| *ACC* | Forward | TTCCAGGCACAGTCCTTAGG | NM_001114269 | 161 |
|  | Reverse | TCATCCAACACGAGCTCAGT |  |  |
| *CPT-*1 | Forward | CTGCCTGACCTATGAAGCCT | NM_001007191. | 221 |
|  | Reverse | AGAAGAGGTGCCTGTCGATC |  |  |
| *FAS* | Forward | CTGCTGAAGCCTAACTCCTCG | NM_001099930 | 242 |
|  | Reverse | TGCTCCTGCACGTCTCCC |  |  |
| *HSL* | Forward | GCAGCATCTTCTTCCGCACA | NM_214315 | 195 |
|  | Reverse | AGCCCTTGCGTAGAGTGACA |  |  |
| *LPL* | Forward | CTCGTGCTCAGATGCCCTAC | NM_214286 | 148 |
|  | Reverse | GGCAGGGTGAAAGGGATGTT |  |  |
| *PPAR*γ | Forward | AGGGCCAAGGATTCATGACA | XM_013981982 | 248 |
|  | Reverse | GTGGTTCAACTTGAGCTGCA |  |  |
| *GAPDH* | Forward | AAGGAGTAAGAGCCCCTGGA | NM_001206359 | 140 |
|  | Reverse | TCTGGGATGGAAACTGGAA |  |  |

Note: *ACC*, acetyl-coA carboxylase; *CPT*-1, carnitine palmitoyl transferase-I; *FAS*, fatty acid synthase; *GAPDH*, glyceraldehyde-3-phosphate dehydrogenase; *HSL*, hormone-sensitive lipase; *LPL*, lipoprotein lipase; *PPAR**γ*, peroxisome proliferator-activated receptor γ.

**Table S3.** Mean count of raw sequences and OTUs among the 4 intervention groups

| Items | BD group | Leu group | Leu_Arg group | Leu_Glu group |
| --- | --- | --- | --- | --- |
| Effective sequences | 45,278 | 38,018 | 37,027 | 37,917 |
| OTUs | 971 | 918 | 919 | 969 |
